# Brightened Optical Transition Hinting to Strong Spin‐Lattice Coupling in a Layered Antiferromagnet

**DOI:** 10.1002/advs.202408343

**Published:** 2025-02-14

**Authors:** Volodymyr Multian, Fan Wu, Dirk van der Marel, Nicolas Ubrig, Jérémie Teyssier

**Affiliations:** ^1^ Department of Quantum Matter Physics University of Geneva 24 Quai Ernest Ansermet Geneva CH‐1211 Switzerland; ^2^ Department of Applied Physics University of Geneva 24 Quai Ernest Ansermet Geneva CH‐1211 Switzerland; ^3^ Advanced Materials Nonlinear Optical Diagnostics lab Institute of Physics, NAS of Ukraine 46 Nauky pr. Kyiv 03028 Ukraine

**Keywords:** 2D materials, multiferroic materials, photoluminescence, van der Waals magnets

## Abstract

Two‐dimensional (2D) van der Waals magnets show strong interconnection between their electrical, magnetic, and structural properties. Here, the emergence of a luminescent transition is revealed upon crossing the *Néel* transition temperature of CrPS_4_, a layered antiferromagnetic semiconductor. This luminescent transition occurs above the lowest absorption level. The optical transitions are attributed to excited states of the t_2g_ orbitals of the Cr^3+^ ions, which are influenced by the distortion of the octahedral crystal field. Specifically, the vicinity of the *Néel* temperature, the distortion switches from an anti‐polar to a polar arrangement, thereby not only promoting an additional luminescent pathway but also significantly strengthening the static dipole moment detected by a marked enhancement in the intensity of the second harmonic generation. These results strongly encourage further investigation into the multiferroic properties and potential coupling mechanisms in CrPS_4_.

## Graphical abstract

The study reveals an unreported luminescent transition in CrPS_4_, a 2D antiferromagnetic semiconductor, near its Néel temperature. This transition arises from changes in Cr^3+^ octahedral crystal field distortion, shifting from anti‐polar to polar. It enhances the dipole moment and second harmonic generation intensity, suggesting a multiferroic state below the Néel temperature.

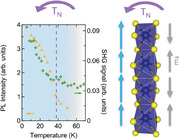

## 1 Introduction

The discovery of magnetism in layered 2D materials offers an unprecedented platform to tailor interactions between spin and charges, potentially leading to the discovery of multiferroic states.^[^ ^1^, ^2^, ^3^ ^]^ The magnetic state of these materials can be modified in the same device by electric field,^[^ ^4^, ^5^ ^]^ doping,^[^ ^6^, ^7^ ^]^ or ultra‐fast optical light pulses,^[^ ^8^, ^9^, ^10^, ^11^, ^12^ ^]^ rather than simply applying an external magnetic field. Such a breakthrough allows for unprecedented control over the physical properties of materials, paving the way for new technological advances. However, progress has been sporadic due to the challenge of identifying effects that demonstrate the coupling between magnetism and electronic structure.^[^ ^13^, ^14^ ^]^ The complexity of electronic structures often leads to unpredictable outcomes in the coupling mechanism with the magnetic state.^[^ ^15^, ^16^, ^17^ ^]^ The case of the antiferromagnet NiPS_3_ illustrates this conundrum, where the observation of narrow emission peaks at *Néel* temperature has sparked long‐standing debates about their magnetic origins.^[^ ^8^, ^18^, ^19^, ^20^, ^21^, ^22^ ^]^ Accurately determining the nature of the ground and excited states within the van der Waals magnet is crucial to unlocking the full potential of 2D van der Waals magnets.

Chromium Thiophosphate (CrPS_4_) has emerged as a promising candidate for tuning the magnetic phase diagram across various thicknesses using an electrostatic gate.^[^ ^7^, ^23^ ^]^ The properties that have drawn significant attention to this material are its air‐stability,^[^ ^23^ ^]^ strong optical response^[^ ^24^, ^25^, ^26^ ^]^ and the increased conduction bandwidth, especially compared to other comparable magnetic van der Waals semiconductors. The latter feature allowed transport studies that revealed not only a new universal mechanism for magnetoconductance,^[^ ^27^ ^]^ but this layered antiferromagnetic compound raised also a lot of interest because of strong proximity effects when brought in contact with other materials^[^ ^28^ ^]^ and unique magnon transport characteristics.^[^ ^29^ ^]^ The optical properties have also been recently revisited,^[^ ^24^, ^30^, ^31^, ^32^, ^33^, ^34^, ^35^, ^36^ ^]^ however, without leading to a definitive picture of the complete electronic structure of the material. For instance, the role of the magnetic transitions on the optical emission and absorption properties remains under debate.^[^ ^24^, ^36^ ^]^

We reveal a luminescent transition in chromium thiophosphate that emerges at higher energies compared to the lowest emission and absorption as the temperature approaches the *Néel* transition. Our spectroscopic analysis over a wide temperature and spectral range indicates that CrPS_4_ exhibits a strong interconnection between lattice, orbital, spin and charge degrees of freedom and optical properties. Key findings are, first, the emergence of luminescence transitions at approximately 2 eV and fano‐like feature at 1.37 eV in the main luminescence continuum favored by a structural rearrangement of chromium environment as long range magnetic order sets‐in; Second, a strong electron‐phonon coupling manifesting as vibronic replica in both Raman and luminescence signals. The optical transitions are attributed to localized *d* − *d*‐transitions between crystal field split states of the Cr^3+^ atoms, affected by the evolution of the distortion of the chromium environment near the *Néel* temperature. This asymmetric distortion along the b‐axis suggests that CrPS_4_ is polar, hinting at the potential for multiferroicity in the material. Our findings not only establish a correlation between magnetism and the chromium ion environment evolution in CrPS_4_, but also enhance our understanding of the electronic ground and excited state configurations of the material, underlining the complex interdependencies that govern its properties.

## 2 Experimental Results

**Figure 1:**
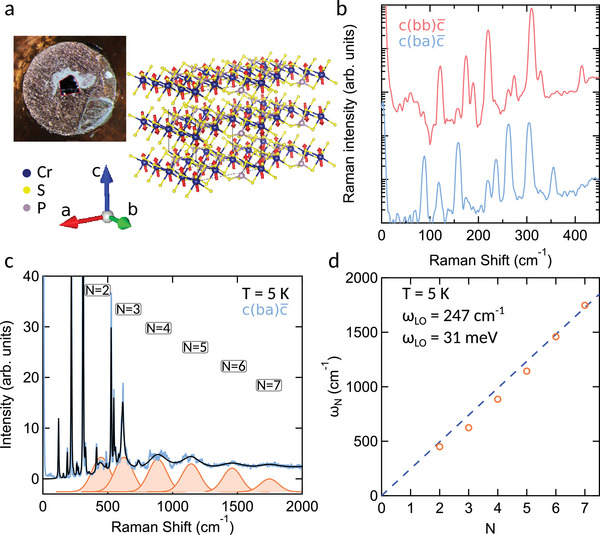
a) Optical micrograph of a representative CrPS_4_ crystal used for this work mounted on the sample holder. On the right, the crystallographic structure is presented. b) Polarization resolved Raman scattering spectra. Polarisation of the excitation laser beam at λ = 532 *nm* is aligned with the b‐axis and the analyzer aligned with the a‐ (blue) and b‐axis (red). c) Broad band spectrum up to 2000 cm^−1^ showing the multi‐phonon character in CrPS_4_. d) Position of the harmonic peaks of the LO mode as a function of mode number. The slope yields a value of 31 meV (247 cm^−1^) of a non‐Raman active LO mode.

We start investigating the optical properties of CrPS_4_ bulk crystals down to low temperatures with a detailed characterization by Raman spectroscopy. We present the crystal structure and an optical micrograph of a bulk crystal of CrPS_4_ in **Figure** **1**a, known to be a layered antiferromagnetic (AFM) semiconductor with a *Néel* temperature at 38 K.^[^ ^30^, ^37^, ^38^, ^39^ ^]^ The Raman modes displayed in Figure 1b agree with the findings of previous reports that allow to determine the complete Raman tensor of the material (see the Experimental Section section for details of the experimental setups used in this work).^[^ ^40^ ^]^ The polarization sensitive peaks enable us to ascertain the precise orientation of the crystallographic axes. The phonon dispersion of CrPS_4_ predicts modes extending up to 450 cm^−1^;^[^ ^41^ ^]^ however, the spectrum exhibit high‐order modes and a periodic broad mode up to energies as high as ≈ 2000 cm^−1^, as shown in Figure 1c. By plotting the energy of each broad mode against its corresponding order, as depicted in Figure 1d, we determine the energy of the fundamental mode to be approximately 31 meV, typically associated to a longitudinal optical (LO) phonon. Similar findings have been reported in related materials like CrI_3_ and transition‐metal monochalcogenides^[^ ^42^, ^43^, ^44^, ^45^, ^46^ ^]^ and are the hallmark of the presence of strong electron‐phonon coupling. The presence of large electron‐phonon coupling is crucial as vibronic states in CrPS_4_ are of primary importance in the interpretation of the optical data which will follow.

**Figure 2:**
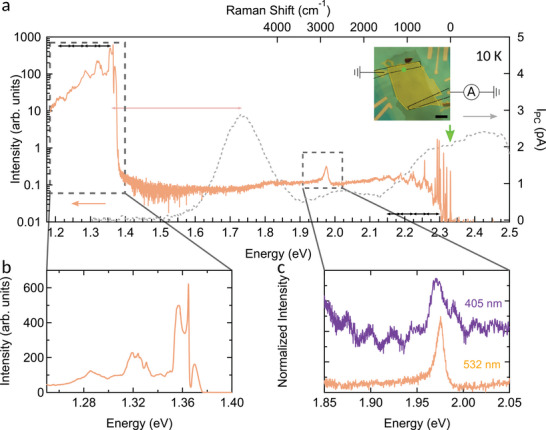
a) Full emission spectrum (solid line) of CrPS_4_ after excitation with a 2.33 eV laser (λ = 532 nm, green arrow). Raman lines are observed close to the excitation energy, while at lower energies the peaks can be attributed to luminescence processes. Dashed grey curve represents the photocurrent of the device shown in the inset, proportional to the absorption of the material (see Supporting Information). Black dashed line represents the thin graphite contacts attached to the sample and the green dot the position at which the spectrum was obtained. The Stokes shift –energy difference between the absorption and the emission lines– is about 350 meV and shown by the pink arrow. Note, as discussed in the text, the electronic states associated to these transition have different nature. b) Zoom‐in of the low energy photoluminescence where the main peak is centered around 1.36 eV. The lower energy peaks are spaced by about 31 meV (also indicated by the arrows in panel (a) which is of the same period than the multi‐phonon peaks observed in the Raman spectrum. c) Comparison of the 2 eV emission spectrum after excitation with 2.33 eV laser (yellow) and 3.06 eV (λ = 405 nm) laser (plum colored curve). Curves are offset for clarity. Oscillations with a period of 30 meV are observed at λ = 405 nm excitation, aligned with vibronic states in the Raman and luminescence spectra, may originate from resonantly excited vibronic states.

We now turn our attention to the broad band emission spectrum ranging from 1.2 to 2.2 eV after exciting the CrPS_4_ crystal with a 2.33 eV (λ = 532 nm) laser at the base temperature of our cryostat (≈10 K, the solid line in **Figure** **2**a). A photoluminescence peak near 2 eV immediately draws attention, yet, to fully capture its origin, we focus first on more discernible features of the spectrum. We identify the Stokes sharp Raman lines discussed earlier at slightly lower energies than the excitation line marked by the green arrow in Figure 2a. In the lowest energy range of the spectrum we detect a pronounced photoluminescence signal centered around 1.35 eV, which is rich in spectroscopic features as seen in Figure 2b.^[^ ^24^, ^34^, ^36^ ^]^ We immediately note that the optical properties of CrPS_4_ diverge significantly from the ones of conventional semiconductors. As shown in Figure 2a, we observe a notable shift between the photoluminescence emission and the main absorption line, the latter obtained through photocurrent measurements (directly linked to the optical absorption,^[^ ^47^ ^]^ as shown in the Supporting Information) performed on a 20 nm thick crystal with graphite contacts (see the inset of Figure 2a) and confirmed by transmission measurements (see Supporting Information, Section S3). A Stokes shift –the difference between the lowest absorption and emission lines in an optical spectrum– is commonly observed in this class of materials, however CrPS_4_ stands out as the value of about 350 meV found here is among the largest reported.^[^ ^48^ ^]^ This finding points toward the fact that the local electrostatic environment is of importance for the electronic and optical properties of the material.

A close inspection of the emission peak centered around 1.35 eV confirms the conclusion and reveals that this photoluminescence displays an oscillating behavior with a period nearly identical to that observed in the Raman data, around 31 meV. This periodicity, coupled with the observed Stokes shift, leads to a Huang–Rhys factor of *S* = 5, signifying very strong electron‐phonon coupling^[^ ^11^ ^]^ within the material confirming the conclusion inferred earlier from the Raman spectroscopy. Additionally, on the high‐energy shoulder of the main photoluminescence peak, a sharp Fano‐like feature at 1.365 eV is detected, corroborating previous reports in this material.^[^ ^24^, ^36^ ^]^ However, as pointed out earlier, the luminescence peak at 1.35 eV intriguingly does not represent the only photoluminescence activity of the material.

In our low‐temperature emission spectrum, we identify an additional peak, albeit weak, centered at 1.97 eV, as illustrated in Figure 2a,c. Remarkably, the energy of this emission peak is higher than the lowest absorption peak of the material. Most likely it is the high position in energy that accounts for the low intensity, which is 3 orders of magnitudes lower than the main photoluminescence peak and of the same intensity as the Raman signal. However, as shown in Figure 2c, upon varying the excitation wavelength of the laser (here from 2.33 to 3.06 eV) the energy of the emission line remains the same, indicating a luminescent nature rather than being the result of a Raman scattering process.

**Figure 3:**
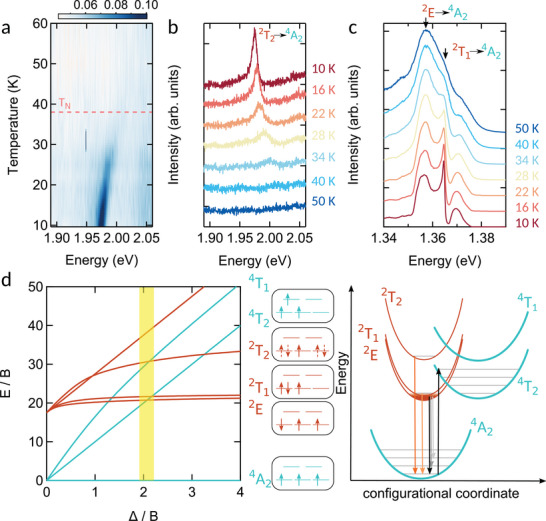
a) False colorplot of the intensity of the 2 eV emission as a function of photon energy and temperature. The AFM transition temperature, *T*_N_ = 38 K, is indicated by the red dashed horizontal line. b) Evolution of the spectrum at selected temperature indicated in the legend of the figure. c) Evolution of the spectrum at selected temperature indicated in the legend of the panel for the low energy photoluminescence, showing the emergence of a Fano‐like feature at 1.365 eV. d) Tanabe‐Sugano fan‐chart (left) and the configurational orbital bands (center) and coordinate diagram (right) of the 3d Cr^3+^. The crystal field strength of CrPS_4_ has been estimated to be Δ/B ≈ 2^[^ ^36^ ^]^ assigning the ^2^E → ^4^A_2_ transition of ≈ 1.35 eV to E/B ≈ 20. Following this assignment the ^2^T_2_ → ^4^A_2_ transition at E/B ≈ 30 corresponds to a photon energy of about 2 eV.

A question that immediately arises is about the physical mechanism behind these optical transitions and the impact of the magnetic properties of CrPS_4_ on its photoluminescence characteristics, especially concerning the Fano feature at 1.365 eV and the newly observed 2 eV transition. Our subsequent analyses, illustrated in **Figure** **3**,b, reveal that this 2 eV transition is indeed appearing around the same temperature as the AFM phase transition occurring in the material. Interestingly, we note that the temperature dependence of the Fano‐like resonance observed at 1.365 eV follows a similar trend, i.e., they both emerge as the temperature approaches the *Néel* temperature, as seen in Figure 3c. This suggests that both transitions are activated by the entrance into the AFM state. The relationship between magnetism and the optical characteristics of CrPS_4_ is subject of debate.^[^ ^24^, ^36^ ^]^ Nevertheless, the significant Stokes shift and pronounced electron‐phonon coupling, combined with a single particle band gap of about 2.3 eV (see Supplementary Information, Section S3, and ref. [31, 49]), lead us to the conclusion that the optical transitions ranging between 1.3 and 2 eV originate from localized *d* − *d*‐transitions on the Cr^3+^ atoms. We stress that optical spectroscopy and tunneling spectroscopy provide complementary information on the electronic structure. *d* − *d*‐excitations are collective modes which can be detected with optical techniques. In contrast, photo‐emission and tunneling spectroscopy probe the single electron band structure.

## 3 Interpretation and Discussion

Understanding the origin of the 2 eV transition necessitates to consider the energy diagram of the Cr^3+^states in an octahedral environment depicted by the Tanabe–Sugano fan‐chart shown in Figure 3d (alongside the configurational orbital bands and coordinate diagram) in the present the *d*3 configuration.^[^ ^50^, ^51^ ^]^ Tanabe–Sugano diagrams are used in coordination chemistry to predict optical transition and present the energy of an orbital state (normalized by the Racah parameter, B, accounting for electron‐electron interactions) as a function of the strength of crystal field. At first glance, this interpretation seems contradicting recent reports of electron transport in CrPS_4_^[^ ^7^, ^27^ ^]^ which imply a significant Cr‐S hybridization leading to a dispersive conduction bandwidth, although some of the valence bands are weakly dispersing.^[^ ^41^, ^49^ ^]^ More important is that the *d* − *d*‐excitations are, by virtue of being many‐body entangled states, rather robust in the presence of dispersion of the single electron bands. As we will discuss later, the CrPS_4_ are not perfect octahedra, which causes a splitting of the multiplets shown in Figure 3. From the bandstructure calculations^[^ ^7^ ^]^ we can estimate these splittings to be of order of tens of meV. While being mindful of those refinements, Figure 3d provides an initial insight into the underlying mechanisms.

At room temperature, absorption and emission processes are well described by spin‐allowed transitions of electrons from the ^4^A_2_ ground state to the ^4^T_2_state. As a result of the on‐site Coulomb and exchange interactions, the eigenstates of 3 electrons in the same 3d shell are in most cases not described by simply putting each of these electrons in an orbital state but require a linear combination of several Slater determinants (see Supporting Information for details). Upon cooling down CrPS_4_ and due to substantial electron–phonon coupling, electrons optically excited to the ^4^T_2_ state undergo intersystem crossing (ICS) with the ^2^E state at about 150 K, shifting the broad luminescence peak from 1.1 eV (^4^T_2_ to ^4^A_2_) observed at 300 K to an emission with central energy around 1.35 eV (^2^E to ^4^A_2_) at low temperatures.^[^ ^34^ ^]^ Note that the orbital occupation of the ground state (^4^A_2_) and the excited ^2^E is identical and their energy difference is due to the on‐site exchange interaction. The oscillations on the lower energy shoulder stems from transition into higher lying vibrational states of the ground state (see the configurational coordinate diagram in Figure 3d). Prior estimations of crystal field splitting,^[^ ^36^ ^]^ represented by Δ/*B* (where *B* is the Racah parameter including electron‐electron interactions), suggest a ratio of about 2, highlighted in Figure 3d by the yellow shaded area. Following this estimate of the crystal field strength we find that the emission energy of the 2 eV peak almost perfectly matches the ^2^T_2_ energy level, while the Fano‐like resonance originates from the ^2^T_1_ energy level. However, as can be seen from the orbital configuration depicted in Figure 3e, transitions from the ^2^T states are spin‐forbidden in the crystal field of an ideal octahedron, which indicates that around the AFM transition changes occur in the Cr^3+^ environment.

**Figure 4:**
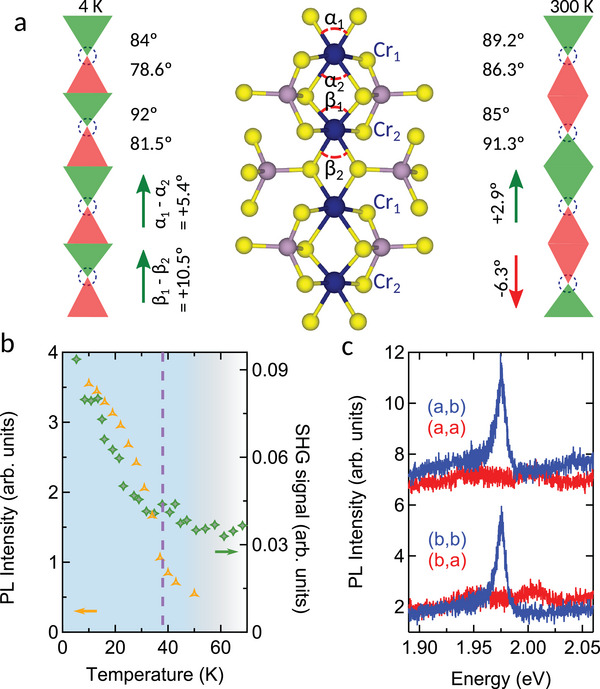
a) Top‐view of the crystal structure of CrPS_4_ showing the distortion angle between the S‐Cr‐S bonds (α_*i*_, β_*i*_ with *i* = 1, 2) in the basal plane at 4 K (left) and 300 K. The arrows show the polarity of the distortion which changes when going to low temperatures. For a given chromium site, green and red triangles represent larger and smaller S‐Cr‐S angles respectively. b) Temperature dependence of the intensity of the photoluminescence 2 eV peak (yellow, left axis) and the intensity of the SHG (green, right axis). We notice a nearly perfect (≈94%) correlation between these quantities. c) Polarization resolved photoluminescence of CrPS_4_ showing extremely strong polarization of the transition along the b‐axis regardless of the polarization of the incident laser.

A detailed examination of the crystal field environment surrounding the Cr^3+^ ions presented in **Figure** **4**a sheds light on a possible mechanism behind the enhancement of these transitions. In fact, each Cr^3+^ (S = 3/2) ion is coordinated by six sulfur atoms in the form of a slightly distorted octahedron as illustrated by the difference in the S–Cr–S bond angle (α_1_ − α_2_ and β_1_ − β_2_ for two adjacent Cr_1_ and Cr_2_ sites, respectively).^[^ ^49^ ^]^ Comparison between high temperature^[^ ^30^, ^39^ ^]^ and low temperature structures^[^ ^37^ ^]^ reveal a transition from anti‐polar to polar alignment of the octahedra as temperatures drop, illustrated in Figure 4a. This configurational change—coinciding with the abrupt elongation of the b‐axis below the *Néel* transition temperature^[^ ^52^ ^]^– is signaled by an enhancement of the SHG intensity as the temperature approaches the *Néel* transition as shown in Figure 4b. An excellent correlation is observed between the SHG signal and the photoluminescence intensity of the 2 eV transition as a function of temperature. The fully linear polarized photoluminescence along the crystallographic b‐axis (see Figure 4c), i.e., parallel to the polar moment, is a clear indication that also the optical transition correlates with the Cr^3+^ site distortion. The modified distortion, albeit being relatively small, of the high temperature octahedral arrangement can lift further the degeneracy of the wavefunctions of the excited state and reinforce transitions that are initially too weak to be detected through strong mixing of the constituent wavefunctions of the multiplets (see Supporting Information).^[^ ^53^ ^]^ Although it does not account for the chromium site distortion, the energy scale of the Tanabe‐Sugano diagram remains however a good approximation. Alternatively, the brightening of the 2 eV transition might be attributed to individual Cr^3+^ sites acting as quantum emitters, shifting from out‐of‐phase emission at higher temperatures, to in‐phase at lower temperatures, following the polar‐distortion long range arrangement. Further investigation is required to fully understand the precise octahedral distortion and its correlation to the non‐linear dielectric tensor. However, the observable changes in the polar environment, as indicated by an enhancing SHG, act as a trigger to the appearance of the ^2^T_1_ and ^2^T_2_ transitions.

The modification in the configuration of the distorted octahedral form is likely to induce an electrical polarization in the layers of CrPS_4_. The increase of the SHG intensity corroborates this conclusion because it is particularly sensitive to inversion symmetry breaking, which for instance arises from electric fields,^[^ ^54^ ^]^ and supported by our symmetry analysis presented in Section S4 (Supporting Information). Note, that neither Raman spectroscopy nor neutron diffraction measurements indicate further symmetry lowering induced by the interlayer antiferromagnetic ordering of CrPS_4_ which excludes that the emerging SHG arises from a similar effect as reported for bilayer CrI_3_.^[^ ^55^ ^]^ Recent estimates on the other hand have inferred a polarization value around 32 pC·m^−2^ at room temperature in the basal plane.^[^ ^25^ ^]^ Following the trends observed in our SHG data and the analyses presented in Figure 4a, we expect an increase of this value at low temperature. While antiferromagnetism of the compound below 38 K is well established, the question of whether the system also exhibits ferroelectric (switchable polarization) or pyroelectric (non‐switchable polarization) characteristics remains open. This potential multiferroicity in CrPS_4_ underscores the complex interplay between its structural, electronic, and magnetic properties, marking it as a material of considerable interest for future research.

In conclusion, we report the brightening of a high‐energy transition within the emission spectrum of the magnetic semiconductor CrPS_4_, which emits at energies above the lowest absorption level and signals the appearance of magnetic order; a rare phenomenon among magnetic materials. We attribute the brightening of the transition to a transformation within the distorted octahedral environment of the Cr^3+^ ions leading to a polar alignment and facilitating spin‐forbidden *d* − *d*‐transitions within the 3d electron configuration. Complementary SHG measurements evidence that the distortion induces polarization within the basal planes of CrPS_4_, particularly below the *Néel* transition temperature, hinting to the existence of multiferroicity in the material. Our results underscore the tight link between lattice dynamics and electronic transition and allow an understanding of the structure of ground and excited state in the material above and below the *Néel* temperature. The disentanglement of relationship between electronic, crystallographic, and magnetic properties of the CrPS_4_ heralds new functional capabilities based on the compound for optoelectronic device applications.

## Conflict of Interest

The authors declare no conflict of interest.

## Supporting information

Supporting Information

## Data Availability

The data that support the findings of this study are openly available in yareta at https://doi.org/10.26037/yareta:2chor6r6w5hilkatkhijy3ryg4.

## References

[1] K. S. Burch , D. Mandrus , J.‐G. Park , Nature 2018, 563, 47. 30382199 10.1038/s41586-018-0631-z

[2] M. Gibertini , M. Koperski , A. F. Morpurgo , K. S. Novoselov , Nat. Nanotechnol. 2019, 14, 408. 31065072 10.1038/s41565-019-0438-6

[3] B. Huang , M. A. McGuire , A. F. May , D. Xiao , P. Jarillo‐Herrero , X. Xu , Nat. Mater. 2020, 19, 1276. 32948831 10.1038/s41563-020-0791-8

[4] S. Jiang , J. Shan , K. F. Mak , Nat. Mater. 2018, 17, 406. 29531370 10.1038/s41563-018-0040-6

[5] B. Huang , G. Clark , D. R. Klein , D. MacNeill , E. Navarro‐Moratalla , K. L. Seyler , N. Wilson , M. A. McGuire , D. H. Cobden , D. Xiao , W. Yao , P. Jarillo‐Herrero , X. Xu , Nat. Nanotechnol. 2018, 13, 544. 29686292 10.1038/s41565-018-0121-3

[6] S. Jiang , L. Li , Z. Wang , K. F. Mak , J. Shan , Nat. Nanotechnol. 2018, 13, 549. 29736035 10.1038/s41565-018-0135-x

[7] F. Wu , M. Gibertini , K. Watanabe , T. Taniguchi , I. Gutiérrez‐Lezama , N. Ubrig , A. F. Morpurgo , Adv. Mater. 2023, 35, 2211653. 10.1002/adma.202211653 37098224

[8] C. A. Belvin , E. Baldini , I. O. Ozel , D. Mao , H. C. Po , C. J. Allington , S. Son , B. H. Kim , J. Kim , I. Hwang , J. H. Kim , J.‐G. Park , T. Senthil , N. Gedik , Nat. Commun. 2021, 12, 4837. 34376692 10.1038/s41467-021-25164-8 PMC8355133

[9] P. Zhang , T.‐F. Chung , Q. Li , S. Wang , Q. Wang , W. L. B. Huey , S. Yang , J. E. Goldberger , J. Yao , X. Zhang , Nat. Mater. 2022, 21, 1373. 36109674 10.1038/s41563-022-01354-7

[10] M. Daabrowski , S. Guo , M. Strungaru , P. S. Keatley , F. Withers , E. J. G. Santos , R. J. Hicken , Nat. Commun. 2022, 13, 5976. 36216796 10.1038/s41467-022-33343-4 PMC9551086

[11] E. Ergeçen , B. Ilyas , D. Mao , H. C. Po , M. B. Yilmaz , J. Kim , J.‐G. Park , T. Senthil , N. Gedik , Nat. Commun. 2022, 13, 98. 35013277 10.1038/s41467-021-27741-3 PMC8748959

[12] M. Matthiesen , J. R. Hortensius , S. Mañas‐Valero , I. Kapon , D. Dumcenco , E. Giannini , M. Łiškins , B. A. Ivanov , H. S. van der Zant , E. Coronado , A. B. Kuzmenko , D. Afanasiev , A. D. Caviglia , Phys. Rev. Lett. 2023, 130, 076702. 36867817 10.1103/PhysRevLett.130.076702

[13] I. Pollini , G. Spinolo , J. Phys. C: Solid State Phys. 1974, 7, 2391.

[14] P. Day , A. Dinsdale , E. R. Krausz , D. J. Robbins , J. Phys. C: Solid State Phys. 1976, 9, 2481.

[15] S. Son , Y. Lee , J. H. Kim , B. H. Kim , C. Kim , W. Na , H. Ju , S. Park , A. Nag , K.‐J. Zhou , Y.‐W. Son , H. Kim , W.‐S. Noh , J.‐H. Park , J. S. Lee , H. Cheong , J. H. Kim , J.‐G. Park , Adv. Mater. 2022, 34, 2109144. 10.1002/adma.202109144 34936713

[16] C. A. Occhialini , Y. Tseng , H. Elnaggar , Q. Song , M. Blei , S. A. Tongay , V. Bisogni , F. M. F. de Groot , J. Pelliciari , R. Comin , Phys. Rev. X 2024, 14, 031007.

[17] D. Lebedev , J. T. Gish , E. S. Garvey , T. W. Song , Q. Zhou , L. Wang , K. Watanabe , T. Taniguchi , M. K. Chan , P. Darancet , N. P. Stern , V. K. Sangwan , M. C. Hersam , Adv. Sci. 2024, 11, 2407862. 10.1002/advs.202407862 PMC11481216 39120494

[18] E. J. K. B. Banda , J. Phys. C: Solid State Phys. 1986, 19, 7329.

[19] K. Hwangbo , Q. Zhang , Q. Jiang , Y. Wang , J. Fonseca , C. Wang , G. M. Diederich , D. R. Gamelin , D. Xiao , J.‐H. Chu , W. Yao , X. Xu , Nat. Nanotechnol. 2021, 16, 655. 33707746 10.1038/s41565-021-00873-9

[20] X. Wang , J. Cao , Z. Lu , A. Cohen , H. Kitadai , T. Li , Q. Tan , M. Wilson , C. H. Lui , D. Smirnov , S. Sharifzadeh , X. Ling , Nat. Mater. 2021, 20, 964. 33903748 10.1038/s41563-021-00968-7

[21] D. Jana , P. Kapuscinski , I. Mohelsky , D. Vaclavkova , I. Breslavetz , M. Orlita , C. Faugeras , M. Potemski , Phys. Rev. B 2023, 108, 115149. 10.1038/s41598-024-67356-4 PMC11289482 39080382

[22] W. He , Y. Shen , K. Wohlfeld , J. Sears , J. Li , J. Pelliciari , M. Walicki , S. Johnston , E. Baldini , V. Bisogni , M. Mitrano , M. P. M. Dean , Nat. Commun. 2024, 15, 3496. 38664432 10.1038/s41467-024-47852-x PMC11045826

[23] J. Son , S. Son , P. Park , M. Kim , Z. Tao , J. Oh , T. Lee , S. Lee , J. Kim , K. Zhang , K. Cho , T. Kamiyama , J. H. Lee , K. F. Mak , J. Shan , M. Kim , J.‐G. Park , J. Lee , ACS Nano 2021, 15, 16904. 34661389 10.1021/acsnano.1c07860

[24] P. Gu , Q. Tan , Y. Wan , Z. Li , Y. Peng , J. Lai , J. Ma , X. Yao , S. Yang , K. Yuan , D. Sun , B. Peng , J. Zhang , Y. Ye , ACS Nano 2020, 14, 1003. 31820929 10.1021/acsnano.9b08336

[25] S. N. Neal , K. R. O'Neal , A. V. Haglund , D. G. Mandrus , H. A. Bechtel , G. L. Carr , K. Haule , D. Vanderbilt , H.‐S. Kim , J. L. Musfeldt , 2D Mater. 2021, 8, 035020.

[26] D. Hou , Z. Jiang , R.‐C. Xiao , C. Liu , X. Chang , Y. Liu , Z. Wang , B. Li , X. Liu , X. Hu , W. Ding , J. Hu , X. Luo , Y. Sun , Z. Sheng , Adv. Opt. Mater. 2024, 12, 2400943.

[27] F. Wu , M. Gibertini , K. Watanabe , T. Taniguchi , I. Gutiérrez‐Lezama , N. Ubrig , A. F. Morpurgo , Nano Lett. 2023, 23, 8140. 37610296 10.1021/acs.nanolett.3c02274

[28] R. Wu , A. Ross , S. Ding , Y. Peng , F. He , Y. Ren , R. Lebrun , Y. Wu , Z. Wang , J. Yang , A. Brataas , M. Kläui , Phys. Rev. Appl. 2022, 17, 064038.

[29] D. K. de Wal , A. Iwens , T. Liu , P. Tang , G. E. W. Bauer , B. J. van Wees , Phys. Rev. B 2023, 107, L180403.

[30] R. Diehl , C.‐D. Carpentier , Acta Crystall. Sect. B: Struct. Crystall. Crystal Chem. 1977, 33, 1399.

[31] A. Louisy , G. Ouvrard , D. M. Schleich , R. Brec , Solid State Commun. 1978, 28, 61.

[32] J. Lee , T. Y. Ko , J. H. Kim , H. Bark , B. Kang , S.‐G. Jung , T. Park , Z. Lee , S. Ryu , C. Lee , ACS Nano 2017, 11, 10935. 29068662 10.1021/acsnano.7b04679

[33] Y. Peng , Z. Lin , G. Tian , J. Yang , P. Zhang , F. Wang , P. Gu , X. Liu , C.‐W. Wang , M. Avdeev , F. Liu , D. Zhou , R. Han , P. Shen , W. Yang , S. Liu , Y. Ye , J. Yang , Adv. Funct. Mater. 2022, 32, 2106592.

[34] S. Kim , S. Yoon , H. Ahn , G. Jin , H. Kim , M.‐H. Jo , C. Lee , J. Kim , S. Ryu , ACS Nano 2022, 16, 16385. 36129115 10.1021/acsnano.2c05600

[35] R. A. Susilo , B. G. Jang , J. Feng , Q. Du , Z. Yan , H. Dong , M. Yuan , C. Petrovic , J. H. Shim , D. Y. Kim , B. Chen , npj Quantum Mater. 2020, 5, 1.

[36] M. Riesner , R. Fainblat , A. K. Budniak , Y. Amouyal , E. Lifshitz , G. Bacher , J. Chem. Phys. 2022, 156, 054707. 35135270 10.1063/5.0079298

[37] S. Calder , A. V. Haglund , Y. Liu , D. M. Pajerowski , H. B. Cao , T. J. Williams , V. O. Garlea , D. Mandrus , Phys. Rev. B 2020, 102, 024408.

[38] Y. Peng , S. Ding , M. Cheng , Q. Hu , J. Yang , F. Wang , M. Xue , Z. Liu , Z. Lin , M. Avdeev , Y. Hou , W. Yang , Y. Zheng , J. Yang , Adv. Mater. 2020, 32, 2001200. 10.1002/adma.202001200 32500563

[39] S. L. Bud'ko , E. Gati , T. J. Slade , P. C. Canfield , Phys. Rev. B 2021, 103, 224407.

[40] S. Kim , J. Lee , C. Lee , S. Ryu , J. Phys. Chem. C 2021, 125, 2691.

[41] H. L. Zhuang , J. Zhou , Phys. Rev. B 2016, 94, 195307.

[42] W. Jin , H. H. Kim , Z. Ye , G. Ye , L. Rojas , X. Luo , B. Yang , F. Yin , J. S. A. Horng , S. Tian , Y. Fu , G. Xu , H. Deng , H. Lei , A. W. Tsen , K. Sun , R. He , L. Zhao , Nat. Commun. 2020, 11, 4780. 32963250 10.1038/s41467-020-18627-x PMC7508859

[43] M. V. Klein , S. P. S. Porto , Phys. Rev. Lett. 1969, 22, 782.

[44] R. C. C. Leite , J. F. Scott , T. C. Damen , Phys. Rev. Lett. 1969, 22, 780.

[45] R. Merlin , G. Güntherodt , R. Humphreys , M. Cardona , R. Suryanarayanan , F. Holtzberg , Phys. Rev. B 1978, 17, 4951.

[46] G. B. Osterhoudt , R. Carelli , K. S. Burch , F. Katmis , N. Gedik , J. S. Moodera , Phys. Rev. B 2018, 98, 014308.

[47] R. T. Collins , K. v. Klitzing , K. Ploog , Phys. Rev. B 1986, 33, 4378. 10.1103/physrevb.33.4378 9938888

[48] P. K. Larsen , S. Wittekoek , Phys. Rev. Lett. 1972, 29, 1597.

[49] Y. Ohno , A. Mineo , I. Matsubara , Phys. Rev. B 1989, 40, 10262. 10.1103/physrevb.40.10262 9991571

[50] Y. Tanabe , S. Sugano , J. Phys. Soc. Jpn. 1954, 9, 753.

[51] Y. Tanabe , S. Sugano , J. Phys. Soc. Jpn. 1954, 9, 766.

[52] T. Fąs , M. Wlazło , M. Birowska , M. Rybak , M. Zinkiewicz , L. Oleschko , M. Goryca , u. Gondek , B. Camargo , J. Szczytko , A. K. Budniak , Y. Amouyal , E. Lifshitz , J. Suffczyński , *Adv. Opt. Mater*. n/a, 2402948.

[53] M. Schmidt , Z. Wang , C. Kant , F. Mayr , S. Toth , A. T. M. N. Islam , B. Lake , V. Tsurkan , A. Loidl , J. Deisenhofer , Phys. Rev. B 2013, 87, 224424.

[54] Q. Song , C. A. Occhialini , E. Ergeçen , B. Ilyas , D. Amoroso , P. Barone , J. Kapeghian , K. Watanabe , T. Taniguchi , A. S. Botana , S. Picozzi , N. Gedik , R. Comin , Nature 2022, 602, 601. 35197619 10.1038/s41586-021-04337-x

[55] Z. Sun , Y. Yi , T. Song , G. Clark , B. Huang , Y. Shan , S. Wu , D. Huang , C. Gao , Z. Chen , M. McGuire , T. Cao , D. Xiao , W.‐T. Liu , W. Yao , X. Xu , S. Wu , Nature 2019, 572, 497. 31367036 10.1038/s41586-019-1445-3

