# Supplementary material for: Brightened Optical Transition Hinting to Strong Spin‐Lattice Coupling in a Layered Antiferromagnet
Source: Adv Sci (Weinh). 2025 Feb 14;12(13):2408343. doi: 10.1002/advs.202408343 (PMC11967845; doi:10.1002/advs.202408343)
Supplement: Supplementary file 1 — Supporting Information [file ADVS-12-2408343-s001.pdf]

ADVANCED SCIENCE
Open Access

Supporting Information

for *Adv. Sci.*, DOI 10.1002/advs.202408343

Brightened Optical Transition Hinting to Strong Spin-Lattice Coupling in a Layered Antiferromagnet

Volodymyr Multian, Fan Wu, Dirk van der Marel, Nicolas Ubrig and Jérémie Teyssier**

Brightened Optical Transition Hinting to Strong Spin-lattice Coupling in a Layered Antiferromagnet

Volodymyr Multian,[1,2,3] Fan Wu,[1,2] Dirk van der Marel,[1] Nicolas Ubrig,[1] and Jérémie Teyssier[1]

[1]*Department of Quantum Matter Physics, University of Geneva, 24 Quai Ernest Ansermet, CH-1211 Geneva, Switzerland*

[2]*Department of Applied Physics, University of Geneva, 24 Quai Ernest Ansermet, CH-1211 Geneva, Switzerland*

[3]*Advanced Materials Nonlinear Optical Diagnostics lab, Institute of Physics, NAS of Ukraine, 46 Nauky pr., 03028, Kyiv, Ukraine*

CONTENTS

S1. Methods 2

S2. Eigenstates of the 3d orbital states 3

S3. Transmission spectra of $CrPS_4$ 5

S4. Non-linear polarization pattern and symmetry analysis 6

S5. References 7

S1. METHODS

Raman, photoluminescence and Second Harmonic Generation measurement

Raman, photoluminescence (PL) and Second Harmonic Generation (SHG) measurements are performed on a unique instrument. We coupled a pulsed Ti:Saphire laser (Coherent Vitesse 800 nm, 100 fs, 80 MHz) to a commercial Raman spectrometer (Horiba LabRAM HR Evolution) using a patented optical module[1] allowing simultaneous recording of polarization resolved Raman, PL and SHG signals. A mm-size bulk $CrPS_4$ crystal is glued (silver paint) on the cold finger of a He flow cryostat (Konti Micro from CryoVac GmbH) allowing measurements from 4K to 350K. Raman and PL spectra are acquired in confocal configuration with a spectral resolution of 0.3 cm^{-1} (0.04 meV). Laser beams from continuous wave lasers emitting at 405 nm or 532 nm and ultrafast laser at 800 nm are focused on the same spot with sizes ranging from 0.4 to 1.0 μm (FWHM) with window-corrected 63× objective. For Raman/PL excitation lasers, a home-tailored module based on a set of the quarter wave (ThorLabs AQWP05M-600) and half wave (ThorLabs AHWP05M-600) plates mounted in motorized stages is used for continuously tune the polarization from linear to circular. Analysis of the polarization state of Raman and PL signals is achieved placing a Glan-Laser polarizer (ThorLabs GL10, extinction ratio 100 000:1) mounted in the motorized rotation stage, followed by a depolarizer (ThorLabs DPP25-A), at the entrance of the spectrometer. Signal acquisition was performed with LN cooled Si charge coupled device (CCD) array (Horiba Scientific Symphony II). Because the quantum efficiency of a Si CCD varies over such a broad spectral range, we calibrated the detector response using the known emission of a 2800 K black body and applied the correction to the data. The Raman laser power is set to 200 μW to minimize the effect of laser heating. The effect at the focal point from the excitation lasers (Raman and SHG) on the sample temperature is monitored using Raman Stokes/anti-Stokes ratio as implemented in Reffit program[2,3].

SHG signal at 400 nm is collected in epidetection geometry in a separate optical channel using a LN-cooled CCD array (Princeton Instruments). A cascade of 3 band-pass interference filters rejects the excitation laser light (ThorLabs FBH400-10, CWL = 400 nm, FWHM = 10 nm). The signal acquisition is realized in a parallel or crossed configuration to the incidence polarization with the use of a Glan-Thompson polarizer (ThorLabs GTH10M) in front of the CCD. The average power of pump beam is set to 10 mW which

corresponds to peak power density of 150 GW/cm^2. After full polarization analysis presented below, parallel polarization configuration maximizing the signal was used to collect temperature dependence data presented in Figure 4b of the main text. The laser heating is again estimated by Raman-based thermometry performed from the same spot.

Photocurrent. Photocurrent measurements were performed by illuminating the sample with a supercontinuum white light laser passing through a contrast filter, allowing the laser wavelength to be tuned continuously between 400 and 1100 nm, while keeping the power fixed at 50 μW. For these measurements, the sample was placed in the cryostat on a holder mounted on a piezo-electric driven x-y stage, allowing stepping the position of the sample relative to the beam with a precision of 50 nm (Cryovac KONTI). The photocurrent was then measured with home-made low noise voltage and current amplifiers, and read out by digital multimeters. For the photocurrent measurements we fabricated thin layered samples (about 10 nm thickness) with thin graphite electrodes following the procedure described in References[4].

S2. EIGENSTATES OF THE 3d ORBITAL STATES

In the main text figure 3d we sketch the orbital occupation of the ground and excited states of the Cr^{3+}. For the sake of completeness we discuss the nature of their eigenstates. The spectroscopic notation $^{2S+1}X_j$ used here characterizes the many-body eigenstates of the 3 electrons in the Cr^{3+} $3d$-shell in terms of the quantum numbers. For example, if the spins of the 3 electrons are all parallel to each other, their total spin is $S = 3/2$ and the m_S can be $\pm 3/2$ or $\pm 1/2$, amounting to $2S + 1 = 4$ different eigenstates with the same energy. The labels A, E, T indicate the orbital character of the $3d^3$ many-body state. For A there is 1 such state, for E there are 2 and for T 3 degenerate states. The eigenstates for the most relevant many-body states are provided.

In an octahedral environment with infinite crystal field, the only available orbitals are d_{xy}, d_{yz} and d_{zx}. For a system with 3 electrons, in the ground state each electron will occupy a different orbital, and each can have spin up or spin down providing $2 \times 2 \times 2 = 8$ possible configurations. The lowest interaction energy is obtained if the total spin of the electrons is maximal, in the present case $S = 3/2$, providing 4 different spin states. Consequently the

ground state is one of the four ${}^4A$ states, or a linear combination thereof:

$$
\begin{aligned}
|g\rangle &= \sum_{m=-3/2}^{3/2} \alpha_m |{}^4A_2; m\rangle \\
|{}^4A_2; 3/2\rangle &= d^\dagger_{xy\uparrow}, d^\dagger_{yz\uparrow}, d^\dagger_{zx\uparrow}|0\rangle \\
|{}^4A_2; 1/2\rangle &= \sqrt{1/3}\{d^\dagger_{xy\downarrow}, d^\dagger_{yz\uparrow}, d^\dagger_{zx\uparrow} + d^\dagger_{xy\uparrow}, d^\dagger_{yz\downarrow}, d^\dagger_{zx\uparrow} + d^\dagger_{xy\uparrow}, d^\dagger_{yz\uparrow}, d^\dagger_{zx\downarrow}\}|0\rangle \\
|{}^4A_2; -1/2\rangle &= \sqrt{1/3}\{d^\dagger_{xy\uparrow}, d^\dagger_{yz\downarrow}, d^\dagger_{zx\downarrow} + d^\dagger_{xy\downarrow}, d^\dagger_{yz\uparrow}, d^\dagger_{zx\downarrow} + d^\dagger_{xy\downarrow}, d^\dagger_{yz\downarrow}, d^\dagger_{zx\uparrow}\}|0\rangle \\
|{}^4A_2; -3/2\rangle &= d^\dagger_{xy\downarrow}, d^\dagger_{yz\downarrow}, d^\dagger_{zx\downarrow}|0\rangle \qquad (1)
\end{aligned}
$$

If the total spin is 1/2, the interaction energy is higher than in the ground state. This extra interaction amounts to $9B + 3C \sim 2$ eV where B and C are Racah parameters. The corresponding set of eigenstates is

$$
\begin{aligned}
|{}^2E; +, \uparrow\rangle &= \sqrt{1/3}\{d^\dagger_{xy\downarrow}, d^\dagger_{yz\uparrow}, d^\dagger_{zx\uparrow} + e^{i2\pi/3} d^\dagger_{xy\uparrow}, d^\dagger_{yz\downarrow}, d^\dagger_{zx\uparrow} + e^{i4\pi/3} d^\dagger_{xy\uparrow}, d^\dagger_{yz\uparrow}, d^\dagger_{zx\downarrow}\}|0\rangle \\
|{}^2E; -, \uparrow\rangle &= \sqrt{1/3}\{d^\dagger_{xy\downarrow}, d^\dagger_{yz\uparrow}, d^\dagger_{zx\uparrow} + e^{-i2\pi/3} d^\dagger_{xy\uparrow}, d^\dagger_{yz\downarrow}, d^\dagger_{zx\uparrow} + e^{-i4\pi/3} d^\dagger_{xy\uparrow}, d^\dagger_{yz\uparrow}, d^\dagger_{zx\downarrow}\}|0\rangle \\
|{}^2E; +, \downarrow\rangle &= \sqrt{1/3}\{d^\dagger_{xy\uparrow}, d^\dagger_{yz\downarrow}, d^\dagger_{zx\downarrow} + e^{i2\pi/3} d^\dagger_{xy\downarrow}, d^\dagger_{yz\uparrow}, d^\dagger_{zx\downarrow} + e^{i4\pi/3} d^\dagger_{xy\downarrow}, d^\dagger_{yz\uparrow}, d^\dagger_{zx\downarrow}\}|0\rangle \\
|{}^2E; -, \downarrow\rangle &= \sqrt{1/3}\{d^\dagger_{xy\uparrow}, d^\dagger_{yz\downarrow}, d^\dagger_{zx\downarrow} + e^{-i2\pi/3} d^\dagger_{xy\downarrow}, d^\dagger_{yz\uparrow}, d^\dagger_{zx\downarrow} + e^{-i4\pi/3} d^\dagger_{xy\downarrow}, d^\dagger_{yz\uparrow}, d^\dagger_{zx\downarrow}\}|0\rangle \quad (2)
\end{aligned}
$$

In total there exist 20 states where 3 electrons occupy the three d_{xy}, d_{yz} and d_{zx} orbitals. Together ${}^4A$ and ${}^2E$ form the subset of states where each these three orbitals is occupied by precisely 1 electron. The other 12 states have one of the three orbitals empty, one occupied with a single electron and one with 2 electrons of opposite spin. They are grouped in two 6-fold degenerate manifolds labeled ${}^2T_1$ (energy $E_g + 9B + 3C$)

$$
\begin{aligned}
|{}^2T_1; yz, \uparrow\rangle &= \sqrt{1/2}\{d^\dagger_{yz\uparrow}, d^\dagger_{zx\uparrow}, d^\dagger_{zx\downarrow} + d^\dagger_{yz\uparrow}, d^\dagger_{xy\uparrow}, d^\dagger_{xy\downarrow}\}|0\rangle \\
|{}^2T_1; zx, \uparrow\rangle &= \sqrt{1/2}\{d^\dagger_{xy\uparrow}, d^\dagger_{yz\uparrow}, d^\dagger_{yz\downarrow} + d^\dagger_{xy\uparrow}, d^\dagger_{zx\uparrow}, d^\dagger_{zx\downarrow}\}|0\rangle \\
|{}^2T_1; xz, \uparrow\rangle &= \sqrt{1/2}\{d^\dagger_{zx\uparrow}, d^\dagger_{xy\uparrow}, d^\dagger_{xy\downarrow} + d^\dagger_{zx\uparrow}, d^\dagger_{yz\uparrow}, d^\dagger_{xz\downarrow}\}|0\rangle \\
|{}^2T_1; yz, \downarrow\rangle &= \sqrt{1/2}\{d^\dagger_{yz\downarrow}, d^\dagger_{zx\uparrow}, d^\dagger_{zx\downarrow} + d^\dagger_{yz\downarrow}, d^\dagger_{xy\uparrow}, d^\dagger_{xy\downarrow}\}|0\rangle \\
|{}^2T_1; zx, \downarrow\rangle &= \sqrt{1/2}\{d^\dagger_{xy\downarrow}, d^\dagger_{yz\uparrow}, d^\dagger_{yz\downarrow} + d^\dagger_{xy\downarrow}, d^\dagger_{zx\uparrow}, d^\dagger_{zx\downarrow}\}|0\rangle \\
|{}^2T_1; xy, \downarrow\rangle &= \sqrt{1/2}\{d^\dagger_{zx\downarrow}, d^\dagger_{xy\uparrow}, d^\dagger_{xy\downarrow} + d^\dagger_{zx\downarrow}, d^\dagger_{yz\uparrow}, d^\dagger_{xz\downarrow}\}|0\rangle
\end{aligned}
$$

and $^2T_2$ (energy $E_g + 15B + 5C$)

$$\begin{aligned}
|^2T_2; yz, \uparrow\rangle &= \sqrt{1/2}\{d^\dagger_{yz\uparrow}, d^\dagger_{zx\uparrow}, d^\dagger_{zx\downarrow} - d^\dagger_{yz\uparrow}, d^\dagger_{xy\uparrow}, d^\dagger_{xy\downarrow}\}|0\rangle \\
|^2T_2; zx, \uparrow\rangle &= \sqrt{1/2}\{d^\dagger_{xy\uparrow}, d^\dagger_{yz\uparrow}, d^\dagger_{yz\downarrow} - d^\dagger_{xy\uparrow}, d^\dagger_{zx\uparrow}, d^\dagger_{zx\downarrow}\}|0\rangle \\
|^2T_2; xy, \uparrow\rangle &= \sqrt{1/2}\{d^\dagger_{zx\uparrow}, d^\dagger_{xy\uparrow}, d^\dagger_{xy\downarrow} - d^\dagger_{zx\uparrow}, d^\dagger_{yz\uparrow}, d^\dagger_{xz\downarrow}\}|0\rangle \\
|^2T_2; yz, \downarrow\rangle &= \sqrt{1/2}\{d^\dagger_{yz\downarrow}, d^\dagger_{zx\uparrow}, d^\dagger_{zx\downarrow} - d^\dagger_{yz\downarrow}, d^\dagger_{xy\uparrow}, d^\dagger_{xy\downarrow}\}|0\rangle \\
|^2T_2; zx, \downarrow\rangle &= \sqrt{1/2}\{d^\dagger_{xy\downarrow}, d^\dagger_{yz\uparrow}, d^\dagger_{yz\downarrow} - d^\dagger_{xy\downarrow}, d^\dagger_{zx\uparrow}, d^\dagger_{zx\downarrow}\}|0\rangle \\
|^2T_2; xy, \downarrow\rangle &= \sqrt{1/2}\{d^\dagger_{zx\downarrow}, d^\dagger_{xy\uparrow}, d^\dagger_{xy\downarrow} - d^\dagger_{zx\downarrow}, d^\dagger_{yz\uparrow}, d^\dagger_{xz\downarrow}\}|0\rangle \qquad (3)
\end{aligned}$$

We finally mention that the $^4T_1$ and $^4T_2$ states have 2 electrons in the one of the d_{xy}, d_{yz} and d_{zx} orbitals, and 1 electron in d_{z^2} or $d_{x^2-y^2}$. The excitation from ^4A to $^4T_1$ or $^4T_2$ therefore has to overcome the crystal field splitting, in the present case about 1.35 eV.

S3. TRANSMISSION SPECTRA OF $CrPS_4$

In the main text, we derive the absorption spectrum from photocurrent measurements on 20 nm thick devices, based on the direct relation between photocurrent and absorption spectroscopy[5]. To validate this assumption in the presence of d–d-transitions, we measured the transmission spectrum at 10 K and 300 K for several thin crystals, ranging from 40 to 150 nm in thickness (insert in Figure S1c), exfoliated on a sapphire substrate. The transmittance of all three regions is fitted simultaneously using a unique model[3] (Figure S1a-b), which allows to extract the real part of the optical conductivity, σ_1, shown squared in Figure S1c.

The results of the transmission measurements confirm the photocurrent measurements shown in Figure 2c of the main text. In particular, $CrPS_4$ shows no absorption around 1.35 eV (the energy where the strongest photoluminescence is measured), the lowest absorption line is centered around 1.75 eV, and a steep absorption edge is observed above 2 eV. Therefore, we can conclude that the Stokes shift in $CrPS_4$ is about 370 meV as reported in the main manuscript. Furthermore, by performing an Urbach tail analysis (assuming that the band gap in $CrPS_4$ is direct[6]), we determine the single-particle band gap to be $E_g = 2.3$ eV, in agreement with previous reports[7,8].

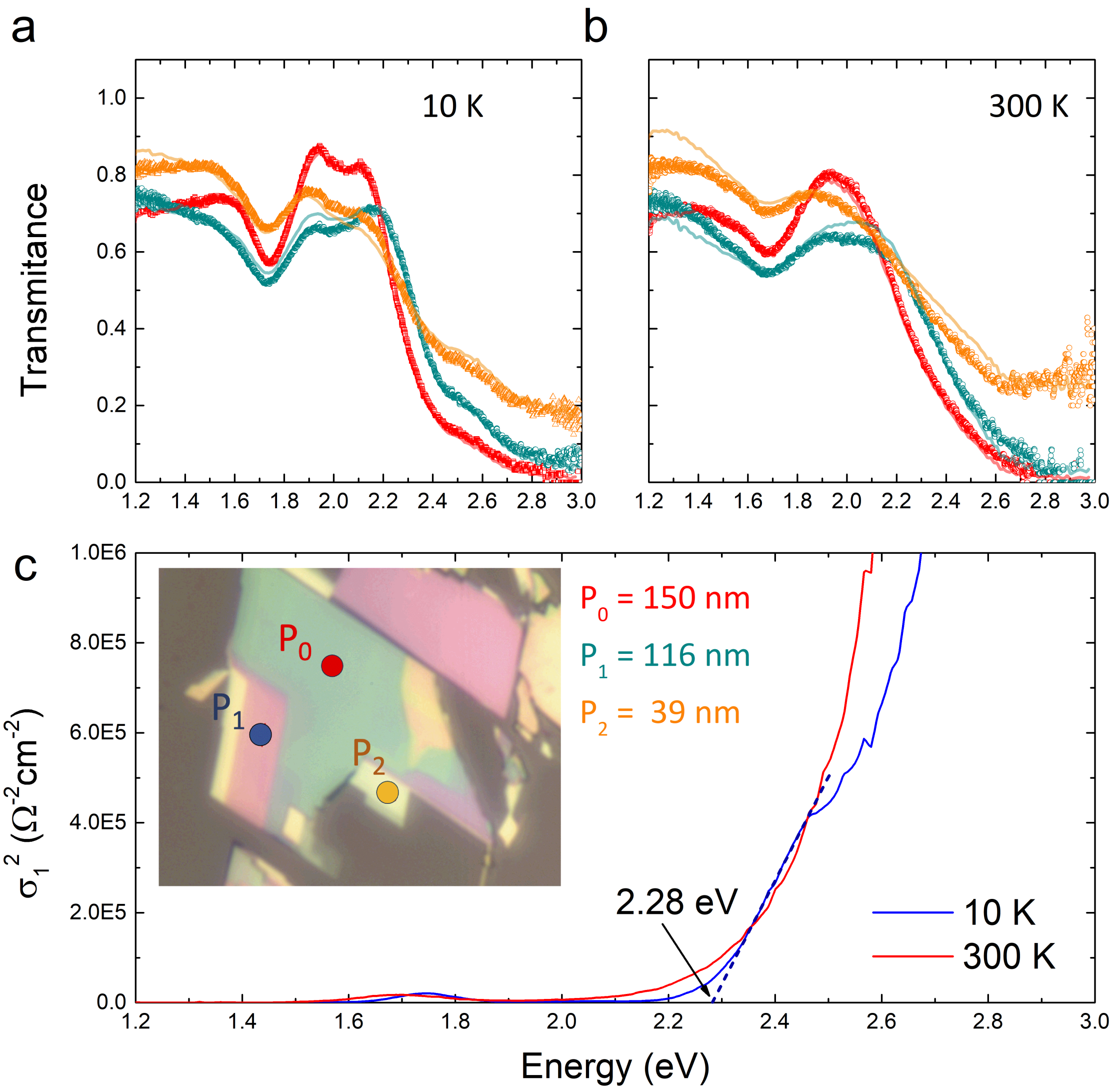

FIG. S1. Experimental transmittance curves (open symbols) for the 3 different areas measured at 10 K (a) and 300 K (a) are fitted (solid lines) simultaneously using a unique model. c) (insert) Areas of transmission measurements on exfoliated $CrPS_4$ crystals of various thicknesses. The crystals are placed on a sapphire substrate .From an Urbach tail analysis on the square or real part of optical conductivity, we estimate the single particle low temperature band gap of $CrPS_4$ at 2.28 eV.

S4. NON-LINEAR POLARIZATION PATTERN AND SYMMETRY ANALYSIS

In the main manuscript we report a strong enhancement of the SHG signal as the temperature is lowered below the *Néel* temperature, indicating a change in the polar distortion of the Cr^{3+} octahedral environment. To elucidate the origin of the increasing SHG, the

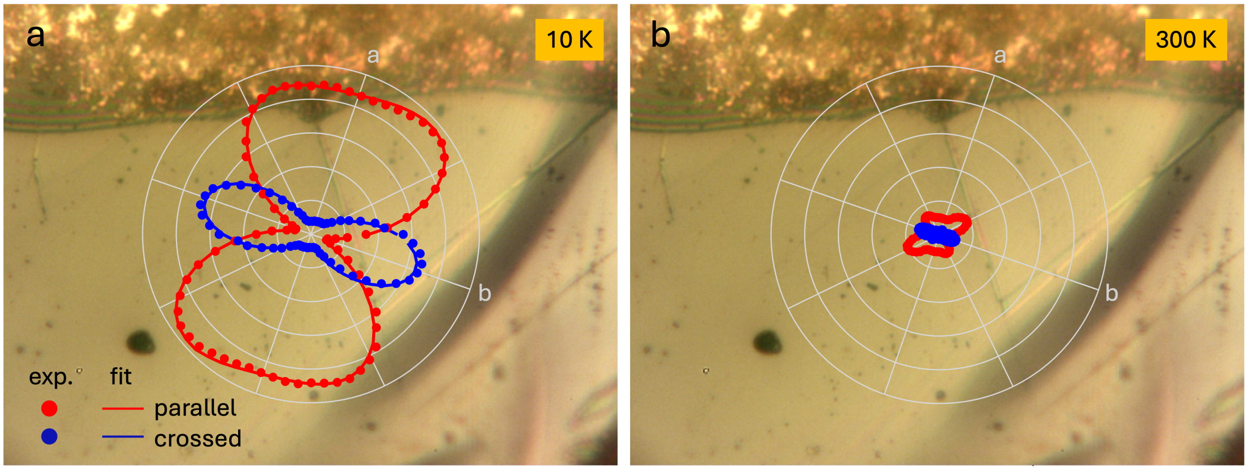

FIG. S2. SHG signal as a function of the incident polarization angle at a) 10 K and b) 300 K on $CrPS_4$ crystal. Polar curves are presented for analyzer parallel (red symbols) and perpendicular (blue symbols) to the excitation polarization. Lines represent a fit considering tensor of the C2 point group symmetry. Orientations of crystallographic *a* and *b*-axis of $CrPS_4$ are shown on polar axis.

polarization dependence in parallel and cross configurations are presented in Figures S2a and S2b , acquired at 10 K and 300 K respectively. Best matching to the experimental data is obtained with a fit considering electric dipole response in a C2 point group symmetry (red curves in Figure S2). The crystal axis are indicated in Figure S2a. Temperature dependence presented in Figure 4b in the main text was measured in a polarization configuration providing the largest SHG signal but shows similar qualitative behavior for all the polarization configurations. This analysis shows that the enhancement of the SHG can be attribute solely to the non-linear dielectric tensor and excludes effects such as a change of the symmetry group due to a structural phase transition, *e.g.*, as reported in CrI_3 bilayers[9], and shows that the non-linear magnetic susceptibility is negligible.

S5. REFERENCES

[1] V. Multian and J. Teyssier, "A beam splitting/mixing module for an optical system and an associated optical system," Patent Pending.

[2] I. Ardizzone, J. Teyssier, I. Crassee, A. B. Kuzmenko, D. G. Mazzone, D. J. Gawryluk, M. Medarde, and D. van der Marel, Physical Review Research **3**, 033007 (2021), publisher:

American Physical Society.

[3] A. B. Kuzmenko, Review of Scientific Instruments **76**, 083108 (2005).

[4] F. Wu, M. Gibertini, K. Watanabe, T. Taniguchi, I. Gutiérrez-Lezama, N. Ubrig, and A. F. Morpurgo, Advanced Materials **35**, 2211653 (2023).

[5] R. T. Collins, K. v. Klitzing, and K. Ploog, Physical Review B **33**, 4378 (1986).

[6] H. L. Zhuang and J. Zhou, Physical Review B **94**, 195307 (2016), publisher: American Physical Society.

[7] A. Louisy, G. Ouvrard, D. M. Schleich, and R. Brec, Solid State Communications **28**, 61 (1978).

[8] Y. Ohno, A. Mineo, and I. Matsubara, Physical Review B **40**, 10262 (1989), publisher: American Physical Society.

[9] Z. Sun, Y. Yi, T. Song, G. Clark, B. Huang, Y. Shan, S. Wu, D. Huang, C. Gao, Z. Chen, M. McGuire, T. Cao, D. Xiao, W.-T. Liu, W. Yao, X. Xu, and S. Wu, Nature **572**, 497 (2019), publisher: Nature Publishing Group.
